# Unilateral pigmentary retinopathy – a review of literature and case presentation

**Published:** 2016

**Authors:** Stamate Alina-Cristina, Burcea Marian, Zemba Mihail

**Affiliations:** * “Prof. Dr. Agrippa Ionescu” Clinical Emergency Hospital, Bucharest, Romania

**Keywords:** unilateral pigmentary retinopathy, unaffected fellow eye, somatic mutation, visual field constriction

## Abstract

Objectives: To report a rare case of unilateral pigmentary retinopathy and describe the clinical and visual field characteristics of this particular case.

Methods: We present the case of a 30-year-old male patient with a gradual loss of the visual field on his left eye (LE) for the past 10 years, with further gradual painless loss of his central visual field in the last year, and no similar symptoms in his right eye. His past medical and ocular history were unremarkable. No family history of acquired or inherited diseases was determined.

Results: Based on the history, clinical findings, and visual field examination, the diagnosis of unilateral pigmentary retinopathy was established. Visual acuity and visual field in the left eye (LE) were severely affected, while in the right eye (RE), they were completely normal.

Conclusions: In this case, distinct features of pigmentary retinopathy were observed only in one eye, with the fellow eye being unaffected. The diagnosis requires a long follow- up period, visual field and electrophysiological testing to rule out a delayed onset of a bilateral form of pigmentary retinopathy.

## Introduction

Pigmentary retinopathy (PR) is a term used to describe a group of inherited, degenerative disorders of the retina, characterized by progressive photoreceptor damage, leading to atrophy, and cell death of the photoreceptors and adjacent layers of the retina. PR primarily affects the rods and consequently the cones, causing blindness in advanced cases, when central retina is involved [**1**].

The prevalence of PR is approximately one in 3.000-4.000 for a total of 1 million affected individuals all over the world [**1**].

Patients can inherit PR in an autosomal- dominant, autosomal-recessive or X-linked recessive pattern, which have all been clearly described and multiple gene defects associated with each inheritance pattern have been defined [**2**].

The initial symptoms of the disease include nyctalopia (night blindness), peripheral visual field constriction, and sometimes loss of the central visual acuity or visual field. The fundus of a patient with PR is characterized by the mottling of the retinal pigment epithelium (RPE), followed by the clumping of the disrupted RPE distributed in bone-spicule formations, attenuated retinal vessels, and waxy pallor of the optic disc [**3**].

There are multiple variants of the classical PR that include unilateral, sector, sine pigmento and punctata albescens PR, which are different both morphologically and electrophysiologically.

Unilateral PR is a rare, sporadic disease, in which the patients have one eye affected by retinal pigmentary degeneration, while the other eye is clinically and functionally normal [**4**,**5**]. This contrasts with the typical forms of PR in which both eyes are affected [**2**].

In order to diagnose unilateral PR, the patient should be monitored for a period of time by means of clinical, perimetric and electroretinographic methods to ensure that the retinal function in the unaffected eye does not alter with time. This method may underline asymmetric bilateral PR in most cases.

The criteria of Francois and Verriest for an authentic case of unilateral pigmentary retinopathy (i.e., idiopathic form) are the following:

• functional changes and fundoscopic appearance typical for a primary pigmentary degeneration must be present in the affected eye;

• symptoms retinal degeneration must be absent in the fellow eye with a normal ERG;

• an inflammatory, infectious, vascular cause in the affected eye must be excluded;

• the period of observation must be long enough (over 5 years) to rule out the possibility of asymmetric inherited PR [**6**].

The etiology of unilateral PR is unknown and is supposed to be the result of a somatic mutation during embryogenesis that affects a percentage of cells in the patient’s body.

Depending on the cells involved, the patient has the possibility to develop this atypical form of unilateral PR during his adult life, if these cells are meant to become the retina and RPE, or might be completely asymptomatic if these cells are destined to become skeletal muscle or bone.

Considering the fact that this particular form of PR appears as a result of a somatic mutation, one might think that the risk of passing along this condition is null, but, if this mutation occurs early enough during embryogenesis, there is a chance of affecting germ line cells and so, a minimal risk of passing along the mutation to offsprings [**3**].

Many conditions can cause a degenerative retinopathy resembling PR and it is imperative to correctly differentiate them from this, because, unlike PR, these etiologies are generally treatable. A high clinical suspicion should be kept, especially when dealing with a unilateral form of PR [**4**,**7**- **13**].

A multitude of etiologies can imitate unilateral PR, amongst them the following being included:

• infection (i.e., congenital rubella, toxoplasmosis, syphilis, Lyme disease);

• inflammation (i.e., retinal vasculitis, old posterior uveitis);

• autoimmunity (i.e., autoimmune retinopathy, cancer-associated retinopathy, acute zonal occult outer retinopathy [AZOOR]);

• trauma (i.e., intraocular foreign bodies, such as siderosis, or blunt trauma, such as severe commotio retinae or retinal detachment);

• drug toxicity (i.e., chloroquine/ hydroxychloroquine, phenothiazines or thioridazine). [**14**].

There is no proven treatment for any form of PR. Various antioxidant, vitamin, and nutritional supplement therapies have been proposed, but with no true benefit for patients with PR. At this point, treatment is supportive and includes low vision aid, genetic testing, counseling, and treatment of associated conditions (cataract or cystoid macular edema) [**3**].

## Methods

We report the case of a 30-year-old male patient who presented to our clinic in February 2015 with gradual visual field loss in his left eye (LE) for the past 10 years, with further loss of his central vision in the last year, without similar symptoms in his right eye (RE). His chief complaint was a reduction of his peripheral left visual field, which required a compensatory left rotation of the head.

**History**

His past medical and ocular history were unremarkable, with no family history of acquired or inherited diseases.

**Ophthalmic examination**

V.A. OD: 20/ 20, V.A. LE: counting fingers, normal IOP, and anterior segment in both eyes.

**Fig. 1:**
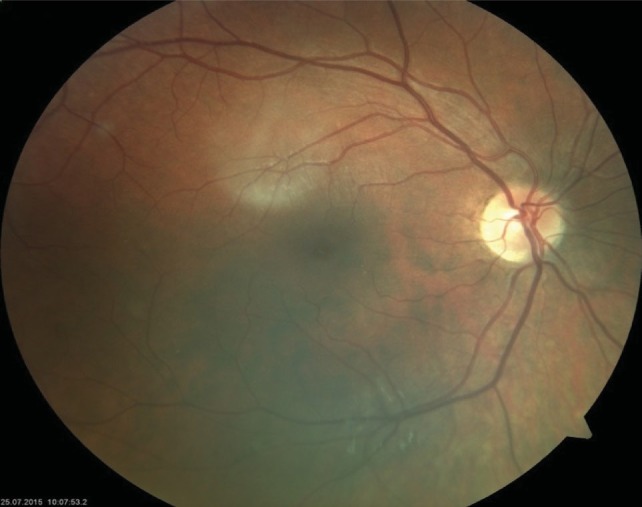
Fundus photograph of the right eye (RE)

**Fig. 2:**
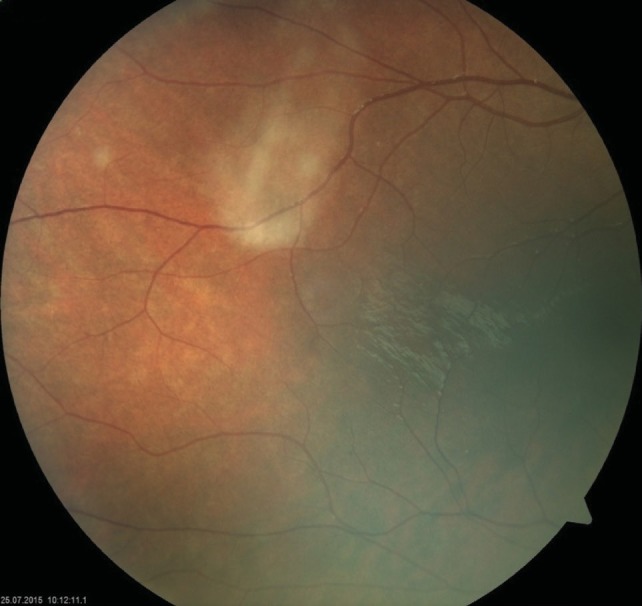
Fundus photograph of the right eye (RE) - periphery

**Fig. 3:**
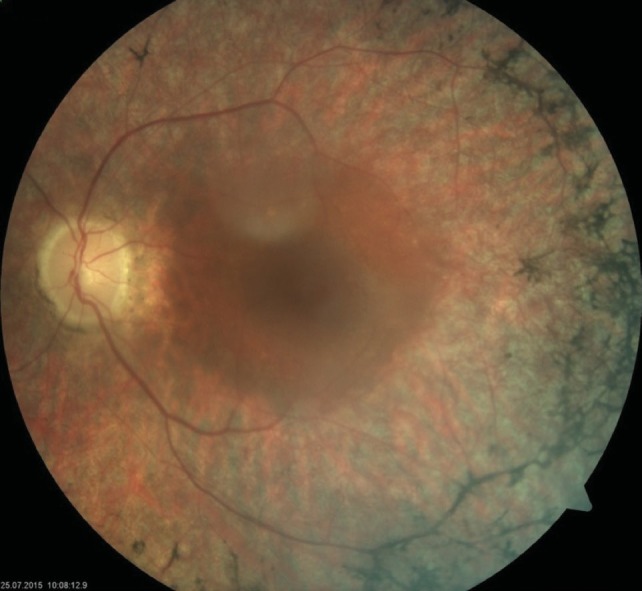
Fundus photograph of the left eye (LE)

**Fig. 4:**
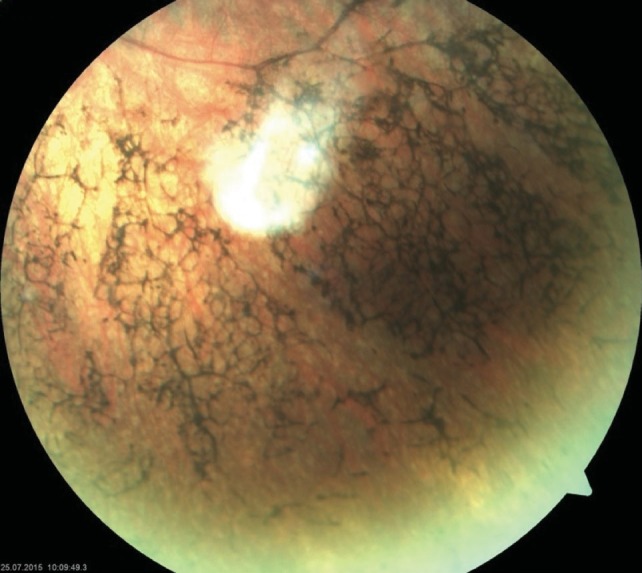
Fundus photograph of the left eye (LE) - periphery

**Fundus examination of the RE was completely** normal, with no pigmentary disturbances (**Fig. 1**,**2**), but that of the LE revealed a waxy disc pallor, markedly attenuated retinal arterioles and clumps of bone-spicule pigments scattered in the mid periphery, in all the quadrants of the retina (**Fig. 3**,**4**).

**Fig. 5:**
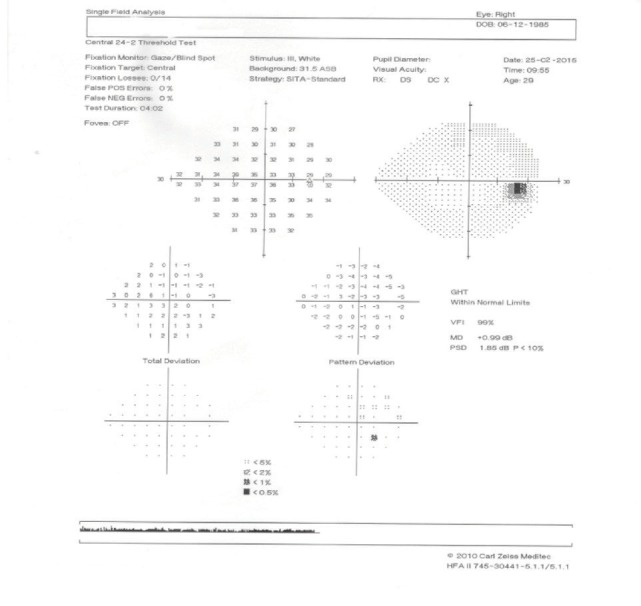
Visual field of the right eye (RE)

**Fig. 6:**
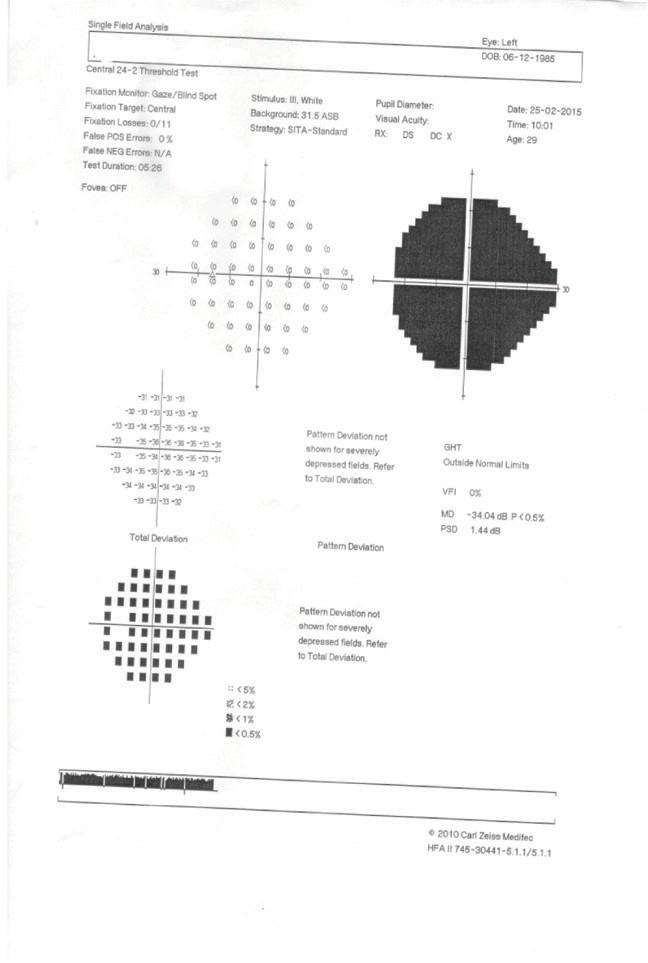
Visual field of the left eye (LE)

**Visual field examination** by a Humphrey perimeter demonstrated a normal visual field in the RE (**Fig. 5**), with generalized sensitivity reduction and severely restricted visual field in the LE (**Fig. 6**).

Electroretinographic testing for this case was not available.

Based on the history of the case, the distinct clinical findings and functional examinations, the patient was diagnosed with unilateral pigmentary retinopathy.

Secondary causes of unilateral PR were excluded. The patient’s history was negative for previous episodes of ocular infections and inflammations, systemic drugs intake, previous trauma or retinal detachment. The serological surveys to rule out syphilis, toxoplasmosis, Lyme disease, were negative. Also, the patient’s mother did not have any history of infection during pregnancy. There was no family history of a similar condition.

Because there was no benefic proven treatment for this condition, and taking into account the severe extent of the disease in this case (an evolution for more than 10 years, that led to a marked reduction of the central visual field, due to the atrophy and cell death of the photoreceptors, and not to an associated macular edema), there was nothing else that could be done than to follow-up the patient, to exclude a delayed onset of an asymmetrical form of bilateral pigmentary retinopathy.

On a following examination in February 2016, the clinical and functional aspects of the first examination were stationary, with no alterations in the fundoscopic appearance and function of the right eye and with the same typical changes for PR in the left eye.

**Fig. 7:**
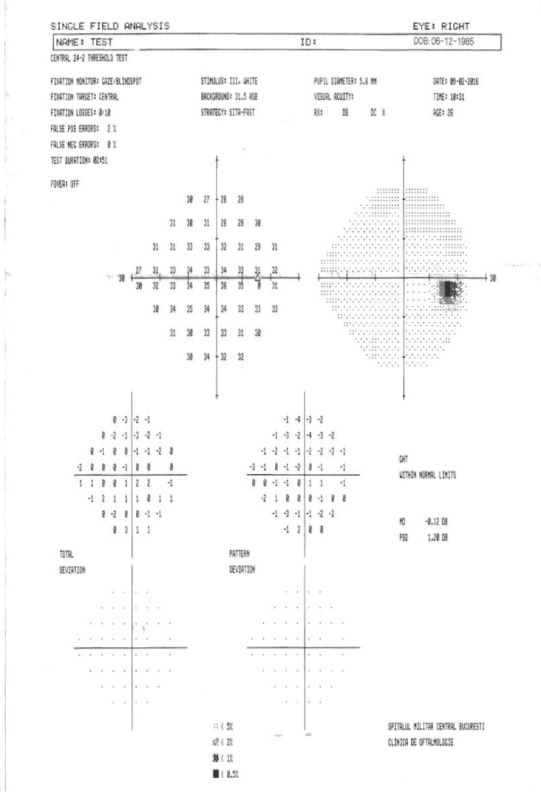
Visual field of the right eye (RE)

**Fig. 8:**
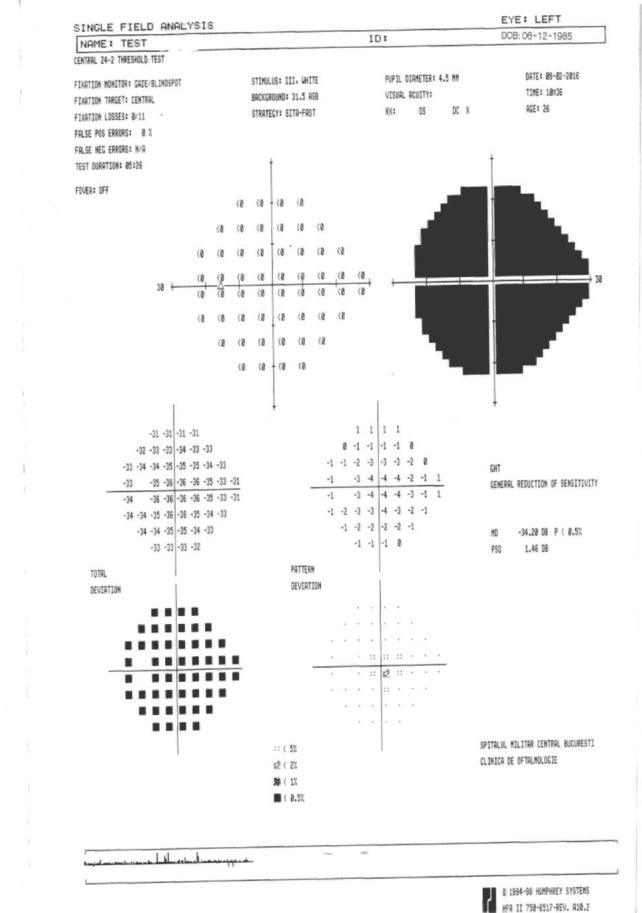
Visual field of the left eye (LE)

The visual filed testing was repeated, still evocating a normal aspect in the RE (**Fig. 7**) and the same generalized sensitivity reduction and severely restricted visual field in the LE (**Fig. 8**).

## Results

Despite the lack of electroretinographic testing, our case nevertheless met 3 out of the 4 Francois and Verriest criteria for an authentic unilateral PR: clinical and functional changes in the affected eye typical for PR, lack of a retinal degeneration in the unaffected eye and exclusion of infectious, inflammatory and vascular causes of retinal pigmentary disturbances [**6**]. The only unfulfilled criterion was a sufficient monitoring period to exclude the onset of a delayed form of bilateral PR (over 5 years). What is important to underline is the fact that although we have only monitored the patient for one year, if we rely on his complaints, we can state that the disease has had a progression for the past 10 years, during which time no alterations appeared in his right eye.

The visual acuity and central visual field in this particular case were severely damaged, due to an extensive period of evolution and progression of this disease, contrasting heavily with an unaffected fellow eye.

## Discussion

Unilateral PR is a rare, sporadic retinal degeneration caused by a somatic mutation during embryogenesis. A true form of unilateral PR is difficult and rare to be diagnose, because, besides the fact that in many cases it proves to be a form of retinal degeneration due to secondary causes, it also requires a long period of follow- up to exclude a bilateral asymmetric form of PR. Therefore, a thorough personal and familial history demonstrates its usefulness in this differential diagnosis.

To conclude, in spite of advances in imaging and testing, PR remains a diagnostic challenge due to its substantial heterogeneity. The same genetic mutation may result in different manifestations in different individuals, while the same manifestation can arise from different mutations [**14**].
